# A Web-Based Prediction Model for Cancer-Specific Survival of Middle-Aged Patients With Non-metastatic Renal Cell Carcinoma: A Population-Based Study

**DOI:** 10.3389/fpubh.2022.822808

**Published:** 2022-02-24

**Authors:** Jie Tang, Jinkui Wang, Xiudan Pan, Xiaozhu Liu, Binyi Zhao

**Affiliations:** ^1^Department of Biostatistics and Epidemiology, School of Public Health, Shenyang Medical College, Shenyang, China; ^2^Department of Urology, Ministry of Education Key Laboratory of Child Development and Disorders, Chongqing Key Laboratory of Pediatrics, China International Science and Technology Cooperation Base of Child Development and Critical Disorders, National Clinical Research Center for Child Health and Disorders (Chongqing), Children's Hospital of Chongqing Medical University, Chongqing, China; ^3^Department of Cardiology, The Second Affiliated Hospital of Chongqing Medical University, Chongqing, China

**Keywords:** nomogram, middle-aged patients, nmRCC, cancer-specific survival, SEER, online application

## Abstract

**Background:**

Renal cell carcinoma (RCC) is one of the most common cancers in middle-aged patients. We aimed to establish a new nomogram for predicting cancer-specific survival (CSS) in middle-aged patients with non-metastatic renal cell carcinoma (nmRCC).

**Methods:**

The clinicopathological information of all patients from 2010 to 2018 was downloaded from the SEER database. These patients were randomly assigned to the training set (70%) and validation set (30%). Univariate and multivariate COX regression analyses were used to identify independent risk factors for CSS in middle-aged patients with nmRCC in the training set. Based on these independent risk factors, a new nomogram was constructed to predict 1-, 3-, and 5-year CSS in middle-aged patients with nmRCC. Then, we used the consistency index (C-index), calibration curve, and area under receiver operating curve (AUC) to validate the accuracy and discrimination of the model. Decision curve analysis (DCA) was used to validate the clinical application value of the model.

**Results:**

A total of 27,073 patients were included in the study. These patients were randomly divided into a training set (*N* = 18,990) and a validation set (*N* = 8,083). In the training set, univariate and multivariate Cox regression analysis indicated that age, sex, histological tumor grade, T stage, tumor size, and surgical method are independent risk factors for CSS of patients. A new nomogram was constructed to predict patients' 1-, 3-, and 5-year CSS. The C-index of the training set and validation set were 0.818 (95% CI: 0.802-0.834) and 0.802 (95% CI: 0.777-0.827), respectively. The 1 -, 3 -, and 5-year AUC for the training and validation set ranged from 77.7 to 80.0. The calibration curves of the training set and the validation set indicated that the predicted value is highly consistent with the actual observation value, indicating that the model has good accuracy. DCA also suggested that the model has potential clinical application value.

**Conclusion:**

We found that independent risk factors for CSS in middle-aged patients with nmRCC were age, sex, histological tumor grade, T stage, tumor size, and surgery. We have constructed a new nomogram to predict the CSS of middle-aged patients with nmRCC. This model has good accuracy and reliability and can assist doctors and patients in clinical decision making.

## Introduction

In recent years, renal cell carcinoma (RCC) incidence has gradually increased, accounting for 2-3% of adult malignant tumors (1). The incidence of RCC in the United States is about 9.1 per 100,000, and the mortality rate is 3.5 per 100,000 (2). It has been reported in the literature that 15% of patients with RCC diagnosed for the first time have developed distant metastases, and another 10-20% of patients with localized RCC eventually develop metastatic RCC (3, 4). The incidence of RCC in men is higher than that in women, about 1.65:1 (5, 6). In 2016, there were 6,700 new diagnoses of RCC in the United States, and 14,240 patients died of renal cancer (7). The prognosis of nmRCC is good, but the 5-year survival rate of metastatic RCC is about 10%, and the median survival time is only 10 months (8). A comprehensive treatment method based on surgery is advocated for localized RCC (9). However, 20-30% of patients with localized RCC still relapse after surgery (10). Therefore, evaluation of the progression, metastasis and prognosis of RCC is critical in clinical management.

At present, studies have shown that clinicopathological factors such as age, sex, and tumor size are related to the prognosis of RCC (11, 12). Guo et al. found that the right RCC has a better prognosis than the left (13). Wang et al. constructed a nomogram to predict the survival of RCC patients with bone metastases and found that age, sex, marriage, tumor histology grade, T stage, N stage, surgery, and radiotherapy are independent risks factors for patients (14). Li et al. developed a nomogram to predict the risk of distant metastasis in patients with RCC (15). Yue et al. found that age is a critical factor in the prognosis of patients with metastatic RCC; elderly patients have a worse prognosis than younger patients (16).

At present, artificial intelligence has been widely used in the medical field. Awais et al. (17) use texture analysis to classify abnormal areas of the mouth and promote the development of oral cancer treatment. Mishra et al. (18) use intelligent drive for multistage assessment of mental disorders to help patients with mental illness. Although various kinds of nomograms have been widely used in clinical practice, the accuracy and specificity of these nomgorams are very worrying. We aimed to establish a specific nomgogram to predict survival in middle-aged patients with renal cell carcinoma. This study used big data based on the Cox regression model to construct a simple nomogram, which is as convenient as possible for users to operate under the premise of ensuring accuracy.

RCC has become significant cancer endangering the health of the population. Accurate prediction of the survival of cancer patients is the key to improving the survival time and quality of life of patients with RCC. At present, using big medical data to establish a prediction model has become an essential means to predict the survival of cancer patients. The nomogram is a user-friendly graphical digital model that can accurately predict the occurrence of a given event based on the numerical estimation of multiple single variables (19). Middle-aged patients with RCC have a good prognosis without distant metastasis. However, accurate prognostic assessment can answer patient consultations and help doctors and patients make clinical decisions. Therefore, we aim to establish a nomogram to predict the CSS of middle-aged patients with nmRCC.

## Patients and Methods

### Data Source and Data Extraction

We downloaded the clinical-pathological data of the patients from the National Cancer Institute's Surveillance, Epidemiology, and Final Results (SEER) project, including patients who were diagnosed with nmRCC in the United States from 2010 to 2018 between 40 and 60 years old. The data of this study can be obtained from the SEER database (http://seer.cancer.gov/). The SEER database is a public database that contains 18 cancer registries and covers ~28% of the American population (20). Patient information can be obtained on the database, including demographic information, tumor characteristics, and survival status. Because the data we used is publicly available, and the patient's personal information is not identifiable, our study did not require ethical approval and informed consent. Our research method complies with the rules of the SEER database.

**Figure 1:**
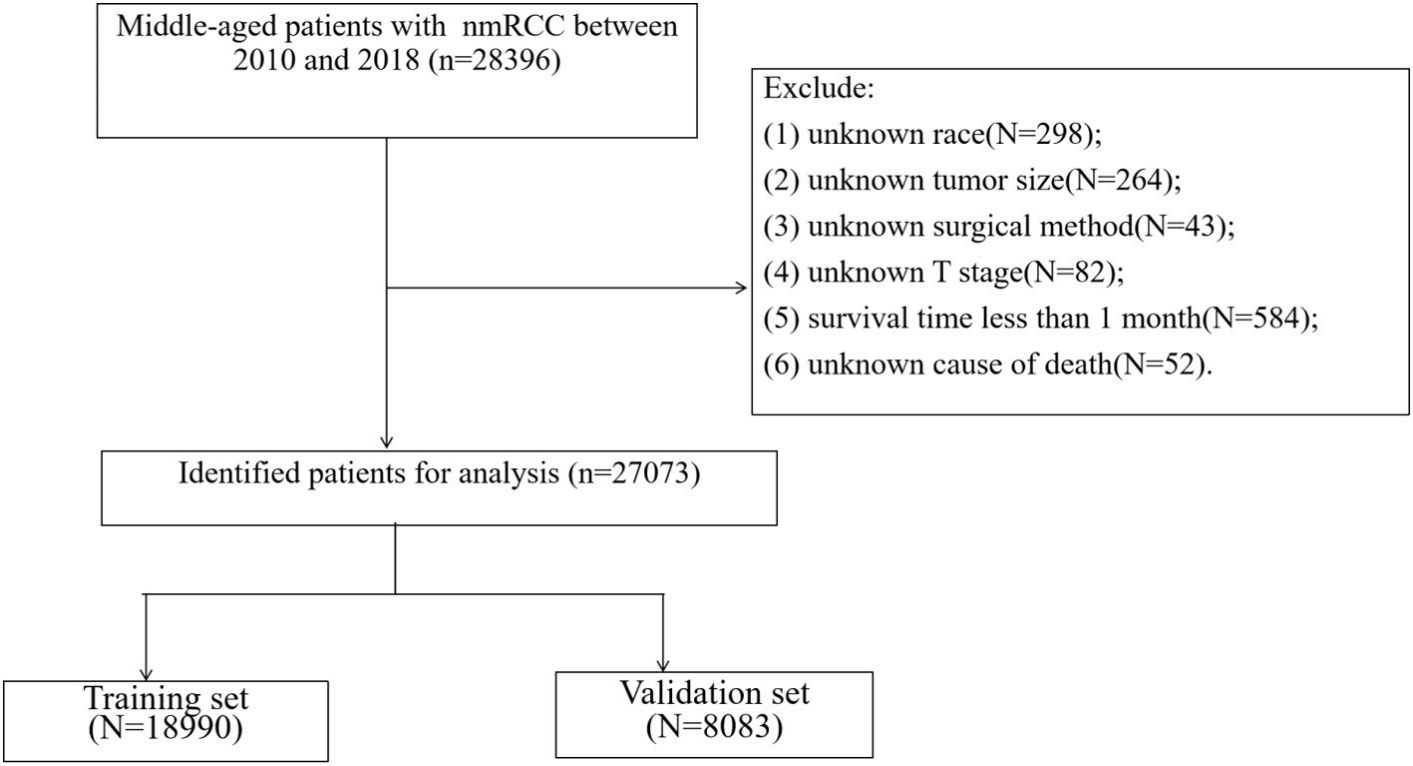
The flowchart of including and dividing patients.

The patient's demographic information and clinical-pathological data include age, sex, race, year of diagnosis, marriage, tumor laterality, tumor histological type, histological grade, T stage, type of surgery, radiotherapy chemotherapy, and survival time. Inclusion criteria: (1) age 40-60 years; (2) pathological diagnosis of renal cell carcinoma (ICD-O-3 codes 8260, 8310, 8312, 8317); (3) diagnosis year 2010-2018. Exclusion criteria: (1) unknown race; (2) unknown tumor size; (3) unknown surgical method; (4) unknown T stage; (5) survival time <1 month; (6) unknown cause of death. The flow chart of patient screening is shown in Figure 1.

The year of diagnosis was divided into 2010-2014 and 2015-2018. The race included white, black, and other races (American Indian/AK Native, Asian/Pacific Islander). Tumor grades have grade I (highly differentiated), grade II (moderately differentiated), grade III (poorly differentiated), and grade IV (undifferentiated). The pathological types of RCC include renal clear cell carcinoma, renal papillary adenocarcinoma, renal chromophobe cell carcinoma, and unclassified renal cell carcinoma. According to the SEER operation code, the operation was divided into local tumor excision (code 10-27), partial nephrectomy (PN, code 30) and radical nephrectomy (RN, code 40-80).

### Univariate and Multivariate Cox Regression Analysis

The patients were randomly divided into a training set (70%) and a validation set (30%). In the training set, univariate and multivariate Cox regression models were used to analyze independent risk factors for survival of nmRCC patients, and the hazard ratio (HR) and 95% confidence interval (CI) were recorded.

### Nomogram Construction for 1-, 3-, and 5-Year CSS

The identified independent risk factors were used to construct a nomogram to predict 1-, 3-, and 5-year CSS in nmRCC patients. All independent risk factors were imported into the nomogram based on the Cox regression model. The risk weights of various variables and the degree of risk are accurately displayed in the nomogram.

### Nomogram Validation

The calibration curve was used to test the accuracy of the prediction model, and we used 1,000 bootstrap samples for internal validation. The 1-, 3-, and 5-year areas under the receiver operating curve (AUC) of the training set and the validation set were used to test the accuracy and discrimination of the prediction model. Similarly, we used the consistency index (C-index) to test the discriminative power of the model.

### Clinical Utility

Decision curve analysis (DCA) is a new calculation method that estimates the net benefits under various risk thresholds to evaluate the clinical value of the model (21). DCA was used to assess the clinical application value of the nomogram and compare it with T staging. In addition, according to the nomogram score, patients were divided into a high-risk group and a low-risk group. Kaplan-Meier curve and log-rank test were used to compare the survival differences of patients in different groups.

### Statistical Analysis

The count data was described by frequency (%), and the chi-square test and non-parametric you test were used to compare groups. Measurement data (age, tumor size) were expressed using mean and standard deviation, and a non-parametric test (*U*-test) was used for differences between groups. The Cox regression model was used to analyze the risk factors of patient survival, and the Kaplan-Meier curve and log-rank test were used to compare the survival differences of patients between groups. All statistical analysis uses SPSS 26.0 and R software 4.1.0. *P*-value <0.05 was considered to be statistically different.

## Results

### Clinical Features

According to the inclusion and exclusion criteria, a total of 27,073 patients were included in the study. These patients were randomly divided into a training set (*N* = 18,990) and a validation set (*N* = 8,083). Table 1 shows the clinicopathological characteristics of all patients. The average age of the patients was 52.4 years, 20,993 patients were white (77.5%), 17,531 patients were male (64.8%), and 16,015 patients were married (59.2%). There were 14,784 (54.6%) patients diagnosed in 2010-2014. Patients with tumor grades I, II, III, and IV was 2,747 (10.1%), 12,412 (45.8%), 6,048 (22.3%), and 928 (3.43%), respectively. 14,571 (53.8%) tumors with T1a stage, 17,534 (64.8%) with the histopathological type of renal clear cell carcinoma, and the average tumor diameter were 46.0 mm. Most patients underwent surgery, 11,750 (43.4%) patients underwent PN, and 13,120 (48.5%) patients underwent RN. Most of the patients did not receive radiotherapy and chemotherapy, 26,702 (98.6%) patients did not receive chemotherapy, and 27,015 (99.8%) patients did not receive radiotherapy. There was no significant difference between the clinical-pathological information of the patients in the training set and the validation set.

**Table 1:** Clinicopathological characteristics of patients with nmRCC.

	**All**	**Training set**	**Validation set**	
	***N*** **= 27,073**	***N*** **= 18,990**	***N*** **= 8,083**	***P***
Age	52.4 (5.61)	52.4 (5.61)	52.4 (5.61)	0.724
Race				0.651
White	20,993 (77.5%)	14,754 (77.7%)	6,239 (77.2%)	
Black	4,288 (15.8%)	2,985 (15.7%)	1,303 (16.1%)	
Other	1,792 (6.62%)	1,251 (6.59%)	541 (6.69%)	
Sex				0.644
Male	17,531 (64.8%)	12,314 (64.8%)	5,217 (64.5%)	
Female	9,542 (35.2%)	6,676 (35.2%)	2,866 (35.5%)	
Year of diagnosis				0.718
2010-2014	14,784 (54.6%)	10,356 (54.5%)	4,428 (54.8%)	
2015-2018	12,289 (45.4%)	8,634 (45.5%)	3,655 (45.2%)	
Marriage				0.152
No	11,058 (40.8%)	7,810 (41.1%)	3,248 (40.2%)	
Married	16,015 (59.2%)	11,180 (58.9%)	4,835 (59.8%)	
Grade				0.143
I	2,747 (10.1%)	1,894 (9.97%)	853 (10.6%)	
II	12,412 (45.8%)	8,797 (46.3%)	3,615 (44.7%)	
III	6,048 (22.3%)	4,196 (22.1%)	1,852 (22.9%)	
IV	928 (3.43%)	651 (3.43%)	277 (3.43%)	
Unknown	4,938 (18.2%)	3,452 (18.2%)	1,486 (18.4%)	
T				0.172
T1a	14,571 (53.8%)	10,178 (53.6%)	4,393 (54.3%)	
T1b	6,105 (22.6%)	4,314 (22.7%)	1,791 (22.2%)	
T2	5,015 (18.5%)	3,557 (18.7%)	1,458 (18.0%)	
T3	1,350 (4.99%)	917 (4.83%)	433 (5.36%)	
T4	32 (0.12%)	24 (0.13%)	8 (0.10%)	
Laterality				0.784
Left	13,090 (48.4%)	9,171 (48.3%)	3,919 (48.5%)	
Right	13,983 (51.6%)	9,819 (51.7%)	4,164 (51.5%)	
Histologic type				0.866
Clear cell	17,534 (64.8%)	12,270 (64.6%)	5,264 (65.1%)	
Papillary	4,011 (14.8%)	2,832 (14.9%)	1,179 (14.6%)	
Chromophobe	1,776 (6.56%)	1,249 (6.58%)	527 (6.52%)	
Not classified	3,752 (13.9%)	2,639 (13.9%)	1,113 (13.8%)	
Tumor size	46.0 (31.9)	46.1 (31.8)	45.7 (32.1)	0.323
Surgery				0.843
No	1,161 (4.29%)	811 (4.27%)	350 (4.33%)	
Local tumor excision	1,042 (3.85%)	742 (3.91%)	300 (3.71%)	
Partial nephrectomy	11,750 (43.4%)	8,222 (43.3%)	3,528 (43.6%)	
Radical nephrectomy	13,120 (48.5%)	9,215 (48.5%)	3,905 (48.3%)	
Chemotherapy				0.266
No/unknown	26,702 (98.6%)	18,740 (98.7%)	7,962 (98.5%)	
Yes	371 (1.37%)	250 (1.32%)	121 (1.50%)	
Radiation				0.531
No/unknown	27,015 (99.8%)	18,952 (99.8%)	8,063 (99.8%)	
Yes	58 (0.21%)	38 (0.20%)	20 (0.25%)	

### Univariate and Multivariate Cox Regression Analysis

All variables were included in univariate Cox regression analysis to screen out survival-related variables. We found that age (HR 1.05, 95%CI 1.03-1.06, *p* < 0.001), sex (HR 0.7, 95%CI 0.59-0.82, *p* < 0.001), tumor histological grade (HR 1.41, 95%CI 1.34-1.49, *p* < 0.001), T stage (HR 2.55, 95%CI 2.35-2.75, *p* < 0.001), tumor size (HR 1.01, 95%CI 1.01-1.01, *p* < 0.001), and surgery (HR 1.23, 95%CI 1.1-1.38, *p* < 0.001) were related to survival prognosis. These factors were included in the multivariate cox regression analysis and showed that all variables were independent prognostic risk factors (Table 2). In other words, these risk factors can be used as factors predicting CSS in patients with nmRCC.

**Table 2:** Univariate and multivariate analyses of CSS in training set.

	**Univariate**			**Multivariate**		
	**HR**	**95%CI**	***P***	**HR**	**95%CI**	***P***
Age	1.05	1.03-1.06	<0.001	1.036	1.021-1.051	<0.001
Race						
White	Reference					
Black	1.075	0.911-1.267	0.391			
Other	0.998	0.774-1.288	0.989			
Sex						
Male	Reference			**Reference**		
Female	0.7	0.59-0.82	<0.001	0.859	0.729-1.013	0.07
Year of diagnosis						
2010-2014	Reference					
2015-2018	0.89	0.73-1.08	0.225			
Marriage						
No	Reference					
Married	0.88	0.76-1.02	0.088			
Grade						
I	Reference			**Reference**		
II	1.238	0.895-1.714	0.198	1.082	0.723-1.618	0.702
III	4.17	3.042-5.717	<0.001	2.468	1.658-3.673	<0.001
IV	14.972	10.711-20.93	<0.001	5.054	3.293-7.759	<0.001
Unknown	3.14	2.26-4.363	<0.001	1.508	0.99-2.297	0.055
T						
T1a	Reference			**Reference**		
T1b	1.982	1.628-2.413	<0.001	1.377	1.065-1.779	0.015
T2	6.509	5.565-7.613	<0.001	3.328	2.612-4.241	<0.001
T3	11.057	8.482-14.412	<0.001	4.861	3.46-6.83	<0.001
T4	74.388	36.566-151.329	<0.001	21.349	9.677-47.103	<0.001
Laterality						
Left	Reference					
Right	0.93	0.81-1.08	0.345			
Histologic type						
Clear cell	Reference					
Papillary	0.909	0.758-1.091	0.308			
Chromophobe	0.366	0.245-0.545	<0.001			
Not classified	1.414	1.209-1.655	<0.001			
Tumor size	1.01	1.01-1.01		1.004	1.003-1.005	<0.001
Surgery						
No	Reference			**Reference**		
Local tumor excision	0.174	0.111-0.273	<0.001	0.254	0.151-0.427	<0.001
Partial nephrectomy	0.08	0.062-0.103	<0.001	0.082	0.059-0.115	<0.001
Radical nephrectomy	0.421	0.345-0.514	<0.001	0.172	0.128-0.232	<0.001

### Nomogram Construction for 1-Year, 3-Year, and 5-Year CSS

**Figure 2:**
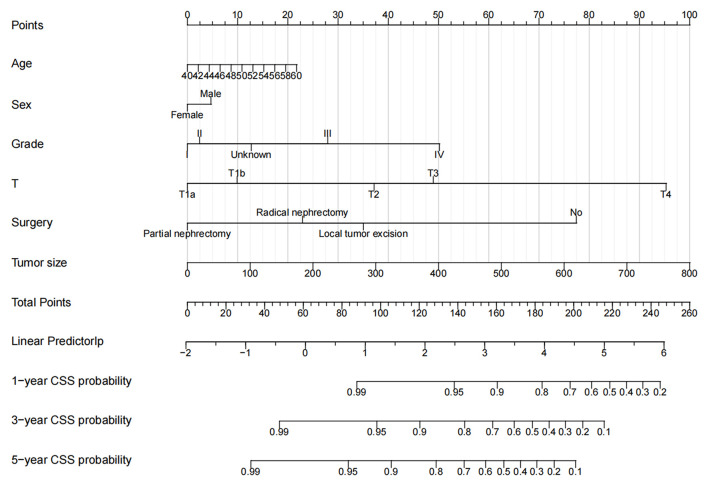
Nomogram for 1-, 3-, and 5-year CSS of middle-aged patients with nmRCC.

Based on the independent risk factors screened out by univariate and multivariate Cox regression analysis, we constructed a new nomogram to predict the 1-year, 3-year, and 5-year CSS of middle-aged patients with nmRCC (Figure 2). The nomogram showed that tumor size and T stage are the most significant factors affecting the patient's CSS, followed by surgery, histological tumor grade, and the final age and sex have little effect on the survival and prognosis of patients.

### Validation of the Nomogram

**Figure 3:**
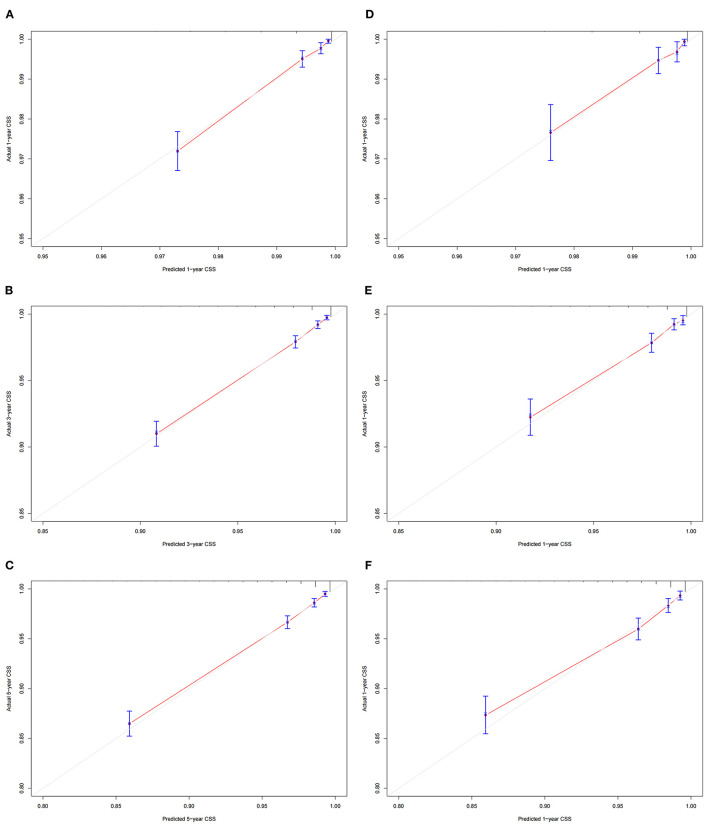
Calibration curves of the nomogram. **(A–C)** For 1-, 3-, and 5-year CSS in the training set; **(D– F)** For 1-, 3-, and 5-year CSS in the validation set.

**Figure 4:**
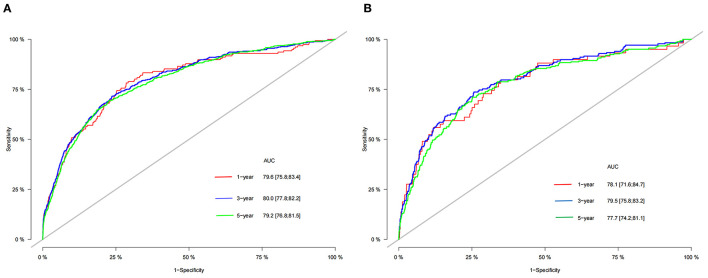
The AUC of nomogram of 1-, 3- and 5-year in the training set **(A)** and validation set **(B)**.

The calibration curve showed that the 1-, 3-, and 5-year predicted values are highly consistent with the actual observed values in the training set, and the validation set are highly compatible with the existing experimental values, suggesting that our model has good accuracy (Figures 3A-F). The C-index in the training set and the validation set were 0.818 (95% CI: 0.802-0.834) and 0.802 (95% CI: 0.777-0.827), respectively, indicating that our prediction model has good discrimination. In the training set, the AUCs of the models that predict patients' 1-, 3-, and 5-year CSS are 0.796, 0.80, and 0.792, respectively (Figure 4A). In the validation set, the AUCs of the models that predict the patient's 1-, 3-, and 5-year CSS are 0.781, 0.795, and 0.777, respectively (Figure 4B). It also proved that the predictive model has good discrimination.

### Clinical Application of the Nomogram

**Figure 5:**
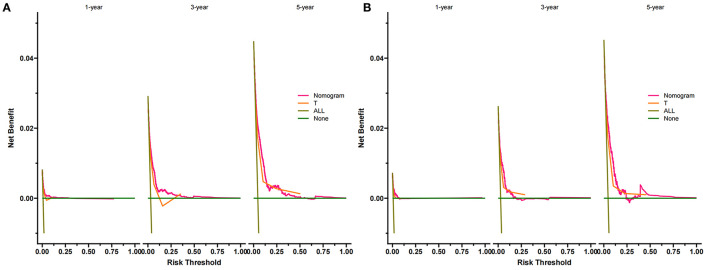
DCA of the nomogram predicting CSS in the training set **(A)** and validation set **(B)**. The Y-axis represents net income, and the X-axis represents threshold probability. The green line means no patients died, and the dark green line means all patients died. When the threshold probability is between 0 and 50%, the net benefit of the model exceeds all deaths or none.

DCA suggested that the nomogram has a better clinical application value in the training and validation set, and it is significantly better than T staging (Figures 5A,B). In addition, we had developed a risk stratification system. According to the score of each patient on the nomogram, all patients were divided into a low-risk group (total score ≤ 72.3) and a high-risk group (total score > 72.3).

**Figure 6:**
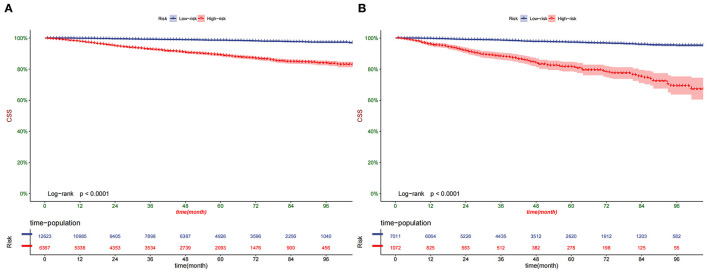
Kaplan–Meier curves of CSS for patients in the low- and high-risk groups in the training set **(A)** and validation set **(B)**.

**Figure 7:**
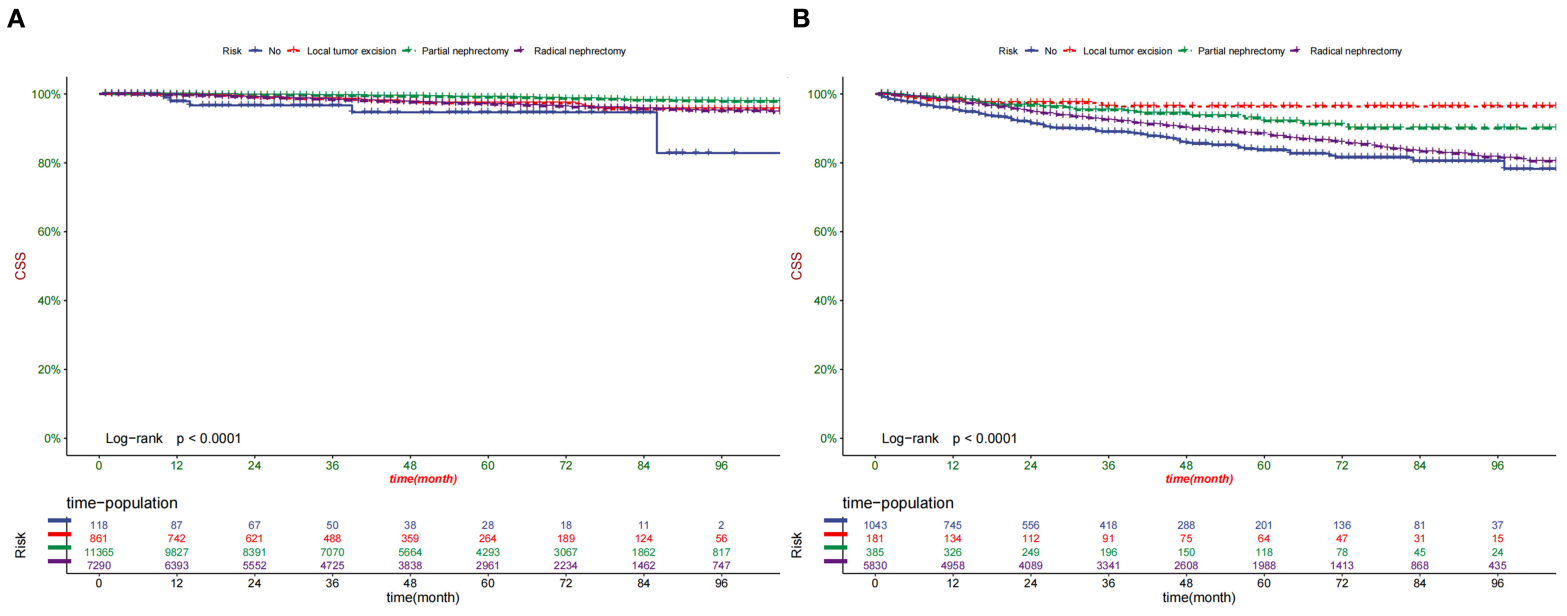
Comparison of different surgical methods of Kaplan–Meier curves in the Low-risk group **(A)** and High-risk group **(B)**.

According to the Kaplan-Meier curve, the high-risk group's 1-, 3-, and 5-year CSS rates were 97.2, 91.5, and 87.2%, respectively. The low-risk group's 1-, 3-, and 5-year CSS rates were 99.7, 98.7, and 97.8%. There was a significant difference in survival between the high-risk group and the low-risk patients in the training and validation set (Figure 6), indicating that our predictive model can accurately identify high-risk patients. In addition, we compared the survival differences of surgical methods in patients with different risk groups. We found that patients with surgery in the low-risk group had a higher survival rate than patients without surgery, including PN, RN, and local tumor excision (Figure 7A). However, although most patients chose RN in the high-risk group, patients with PN and local tumor excision have a higher survival rate than RN (Figure 7B).

### Online Application for CSS Prediction

Based on the nomogram we constructed, we developed a web application to predict the CSS of middle-aged patients with nmRCC. Visit https://xiudanpan.shinyapps.io/DynNomapp/ to enter the website. Enter the patient's clinical characteristics, and we can obtain the CSS of the patient at each time.

## Discussion

RCC is a common tumor of the urinary system in the world, the incidence of women ranks ninth, and the incidence of men ranks seventh (22, 23). Although surgical treatment, immunotherapy, targeted therapy, and other RCC treatment methods are developing rapidly. However, due to the widespread local recurrence, distant metastasis, and drug tolerance of RCC, the prognosis of RCC patients is not very optimistic (24). To improve RCC patients' prognosis and quality of life, more and more renal cancer surgery risk scoring standards and renal cancer prognostic risk stratification have been established (25–27). The prognosis of early RCC is relatively good. The study reported that early asymptomatic RCC prediction is significantly better than that of symptomatic RCC (28). With improved health awareness and the popularization of health examinations, symptomatic kidney cancer is rare, and patients with advanced kidney cancer are more common. The proportion of early asymptomatic kidney cancer is gradually increasing, with reports ranging from 46.2 to 61% (29). It may be because of the recent increase in abdominal imaging, which is the main reason for the early diagnosis of asymptomatic kidney cancer (30). One study found that frequent use of CT for abdominal scans is associated with the risk of nephrectomy (31).

This study focused on middle-aged nmRCC patients and established a prognostic nomogram for predicting the CSS of middle-aged nmRCC patients for the first time. Because middle-aged patients' remaining lives with nmRCC are still very long, an accurate prognosis can help patients improve their survival rate and quality of life. Our constructed nomogram can accurately predict patients' 1-, 3-, 5-year CSS. According to the univariate and multivariate analysis of patients, age, sex, histological grade, T stage, surgery, and tumor size are independent risk factors.

Similar to other studies, our results also found that age is a critical factor in the prognosis of patients, even in middle-aged patients (32). Because the increase of age will bring about the weakening of the immune system, further causing the deterioration of the tumor and reducing the survival time of the patient (33). In our study, men have a higher incidence of kidney cancer and a higher mortality rate. Sex as a prognostic factor of patients may be related to hormone levels in the body, such as androgens and testosterone can cause specific cancers (34, 35).

Previous studies have found that tumor characteristics are also critical factors for patient survival, such as histological tumor grade, T stage, N stage, and distant metastasis (36). Our study found that tumor size and histological tumor grade are independent risk factors for patient prognosis. The histological grade is related to the stemness of the tumor. Previous studies have found that high-grade tumors are related to bladder cancer and prostate cancer (37, 38). Because high-grade tumors are often highly malignant and aggressive tumors. In addition, tumor size is also associated with the patient's prognosis. The larger the tumor, the higher the risk of metastasis and invasion.

The TNM staging system is a standard staging system for all tumors. It is mainly determined by postoperative pathological results and clinical staging (39). According to the patient's tumor condition (T), lymph node (N), distant metastasis (M), the cancer is divided into different stages. Indeed, TNM staging is related to the patient's prognosis. The higher the stage, the worse the patient's prognosis. For nmRCC, there is no lymph node and distant metastasis, and only T staging can reflect the staging of the tumor. Our study found that the T stage is the most critical factor affecting the prognosis of patients. The higher the T stage, the worse the patient's prognosis. This also proved that T staging should be used as an essential component of the nomogram.

Tumor treatment mode is also an important prognostic factor for patients with RCC. Surgery, as the essential treatment method, is the most critical factor for the prognosis of renal cancer patients (40). The nomogram showed that patients with PN have the best prognosis, while those without surgery have the worst prognosis. Our risk stratification system suggested that most patients in the low-risk group choose PN and have a high survival rate. For high-risk patients, most patients choose RN. Although patients with RN and local tumor excision have a higher survival rate, this may be caused by selection bias. Because of more extensive and higher T-stage tumors, doctors and patients are more inclined to choose RN. And these patients will have worse outcomes.

This study used the identifiable variables in the SEER database to construct predictions of 1-, 3-, and 5-year CSS in middle-aged patients with nmRCC. The model has good accuracy and discrimination. The calibration curve of the nomogram indicated that the prediction accuracy of the prediction model is very high. The C-index and AUC of the nomogram are about 0.8, which stated that the discriminative accuracy of the prediction model is about 80% and proved that the model is reliable. This nomogram can predict the prognosis of middle-aged patients with nmRCC and provide a reliable basis for personalized treatment and monitoring.

This study used the identifiable variables in the SEER database to construct predictions of 1-, 3-, and 5-year CSS in middle-aged patients with nmRCC. The model has good accuracy and discrimination. The calibration curve of the nomogram indicated that the prediction accuracy of the prediction model is very high. The C-index and AUC of the nomogram are about 0.8, which stated that the discriminative accuracy of the prediction model is about 80% and proved that the model is reliable. This nomogram can predict the prognosis of middle-aged patients with nmRCC and provide a reliable basis for personalized treatment and monitoring.

This study also has some limitations. First of all, we did not include some possible clinical factors, such as BMI, smoking, drinking, hypertension, genetic markers, etc. But we had included important clinical-pathological information, such as tumor stage, surgery and other vital factors, so our results will not be too biased. Secondly, our study was a retrospective cases study, and there may be some deviations that are difficult to adjust. Further prospective studies are necessary to validate our prediction model. Finally, we only used the data in the SEER database for internal validation, and the subsequent external proof is needed to validate the model's accuracy.

## Conclusion

We found that independent risk factors for CSS in middle-aged patients with nmRCC were age, sex, histological tumor grade, T stage, tumor size, and surgery. We have constructed a new nomogram to predict the CSS of patients. This model has good accuracy and reliability and can assist doctors and patients in clinical decision making.

## Data Availability Statement

Publicly available datasets were analyzed in this study. This data can be found at: https://seer.Cancer.gov/.

## Ethics Statement

The data of this study is obtained from the SEER database. The patients' data is public and anonymous, so this study does not require ethical approval and informed consent.

## Author Contributions

JW, BZ, XL, and JT contributed to the conception and design. JW, BZ, and JT collected and analyzed the data. JW, BZ, XP, and JT drew the figures and tables. JT, XL, XP, and JW wrote the draft. JT, BZ, and XP contributed to manuscript writing and revision. All authors approved the final manuscript.

## Conflict of Interest

The authors declare that the research was conducted in the absence of any commercial or financial relationships that could be construed as a potential conflict of interest.

## Publisher's Note

All claims expressed in this article are solely those of the authors and do not necessarily represent those of their affiliated organizations, or those of the publisher, the editors and the reviewers. Any product that may be evaluated in this article, or claim that may be made by its manufacturer, is not guaranteed or endorsed by the publisher.
